# Urinary TNF-α and NGAL are correlated with the progression of nephropathy in patients with type 2 diabetes

**DOI:** 10.3892/etm.2013.1315

**Published:** 2013-09-26

**Authors:** JIAN WU, YAQING DING, CHUNLING ZHU, XIAOHONG SHAO, XIN XIE, KAN LU, RUI WANG

**Affiliations:** Department of Endocrinology, Shanghai TCM-Integrated Hospital, Shanghai 200082, P.R. China

**Keywords:** diabetic nephropathy, tumor necrosis factor-α, neutrophil gelatinase-associated lipocalin

## Abstract

The aim of this study was to investigate the correlation of the proinflammatory marker tumor necrosis factor-α (TNF-α) and the tubular marker neutrophil gelatinase-associated lipocalin (NGAL) with the progression of the early stage of type 2 diabetic nephropathy (DN). Baseline levels of urinary TNF-α and NGAL were measured in 63 non-diabetic controls and 201 patients with type 2 diabetes and different albuminuria statuses. The patients with diabetes (n=125) with normo- or microalbuminuria were subsequently followed-up for 28 (25–32) months, with routine measurements of creatinine and urinary albumin excretion (UAE). It was observed that baseline levels of urinary TNF-α and NGAL were significantly elevated and correlated with the severity of albuminuria in patients with diabetes. During the follow-up, the urinary levels of TNF-α and NGAL were observed to be significantly correlated with a rapid decline in the estimated glomerular filtration rate (eGFR). Following adjustment for other progression promoters, including albuminuria, TNF-α remained a significant predictor of eGFR decline. These results suggest that inflammation is important in the pathogenesis of DN and indicate that TNF-α may be used as an independent predictor for the progression of DN at the early stage.

## Introduction

Diabetic nephropathy (DN) is a common microvascular complication of diabetes mellitus, as well as the leading cause of renal failure worldwide (1,2). It occurs in approximately one-third of all patients with diabetes and is independently correlated with an increased risk of end-stage renal disease (1,3). The prognosis of DN has been poor due to diagnosis at an advanced stage and the high rate of recurrence. Although significant efforts have been made to develop novel diagnostic and therapeutic approaches, DN remains a severe condition with high rates of morbidity and mortality (1–3). Accordingly, the early diagnosis of DN is critical to prevent the long-term damaging effects of kidney loss in patients with diabetes (4,5).

The pathogenesis of DN is complex and not fully understood. It has been demonstrated that a number of metabolic and hemodynamic perturbations are important in the functional and structural changes of the kidney in DN, which are associated with the development and outcomes of the disease (6). Currently, the dysfunction of the glomerular filtration barrier, which results in increased urinary albumin excretion (UAE), is considered to be an early sign of DN (5–7). As DN progresses, gradual increases in UAE and a decline in renal function may be observed, ultimately leading to end-stage renal disease (2,8). Accordingly, microalbuminuria is considered to be an important prognostic marker for the early detection of DN (5,8–10). However, it has been shown that many patients with diabetes may still develop DN, even if their urinary albumin levels are within the normal range (7,11,12). Moreover, it has also been suggested that albuminuria is not a good early marker, since it is only possible to observe microalbuminuria when significant damage to glomerular function has already occurred (13). Therefore, more sensitive and specific biomarkers are required to detect DN at an earlier stage (5,9).

It has been demonstrated that renal injuries in DN are particularly heterogeneous and that almost all of the cellular elements in a diabetic kidney may be affected (4,5,12). In addition to glomerular dysfunction, tubulointerstitial damage may also be important in the pathogenesis and progression of DN (5,9,14,15). A number of markers of tubular damage have been identified and there is growing evidence to support the use of these markers as early predictors of DN (5,14). Neutrophil gelatinase-associated lipocalin (NGAL) is a member of the lipocalin protein family that is produced in epithelial cells and neutrophils (16,17). Previous studies have shown NGAL to be one of the most significantly upregulated proteins in the kidney tubules following ischemic injury, indicating that it is a sensitive marker for acute kidney injury (AKI) (18–20). Interestingly, in a number of patients with type 1 diabetes mellitus, the urinary NGAL level was already elevated, irrespective of whether micro- or macroalbuminuria was apparent, indicating that tubular damage may occur independently and earlier than glomerular dysfunction (16,17).

Moreover, a number of studies have suggested that inflammation is crucial in promoting the development and progression of DN (5,21,22). Activated by the metabolic and hemodynamic derangements in the diabetic kidney, an inflammatory response may occur at a very early stage of diabetes mellitus (21). This results in several devastating effects, involving the dysregulation of different immune and inflammatory cells, inflammatory cytokines and stress-activated protein kinases (5,21,23). Consistent with this, it has been shown that patients with diabetes who progress to DN exhibit signs of inflammation for years prior to the onset of the disease. Tumor necrosis factor-α (TNF-α) is a proinflammatory cytokine that is essential in the regulation of inflammation, apoptosis and oxidative stress in the kidney (16,23,24). A high level of urinary TNF-α is correlated with renal injury and a disruption of the glomerular permeability barrier in patients with diabetes. This indicates that TNF-α is causally linked to renal injury in patients with diabetes and may be used as an early biomarker for the progression of DN.

Although these observations indicate that elevated levels of NGAL and TNF-α are implicated in kidney injury, it has not yet been elucidated whether NGAL and TNF-α are independently predictive of the onset and progression of DN. Therefore, we performed a prospective observational study to investigate the relationship between different biomarkers and to determine the role of urinary TNF-α and NGAL as predictors of a decline in the estimated glomerular filtration rate (eGFR) in patients with type 2 diabetes. Specifically, we investigated whether, subsequent to adjusting for other clinical factors such as albuminuria, TNF-α and NGAL were able to provide additional prognostic information with regard to the progression of DN at the early stage.

## Materials and methods

### Subjects

A total of 201 patients with diabetes (108 males and 93 females) who had been referred to the Department of Endocrinology of the Shanghai Traditional Chinese Medicine (TCM)-Integrated Hospital (Shanghai, China) between February 2009 and March 2013 were enrolled in this study. This study was approved by the institutional review board of Shanghai TCM-Integrated Hospital, and informed consent was obtained from patients or their families as appropriate. All patients involved in this study fulfilled the following inclusion criteria: age >18 years; initial diagnosis of diabetes at >30 years of age; no sign of renal disease other than DN; no history of cardiovascular disease, including stroke, heart disease and arteriosclerosis and no symptoms of acute inflammatory disease. Patients were stratified according to urine albumin/urine creatinine (Cr) ratio (u-ACR) into three groups: normoalbuminuria (<30 mg/g Cr), microalbuminuria (30–300 mg/g Cr) and macroalbuminuria (>300 mg/g Cr). Baseline u-ACRs levels were consistent in at least two consecutive measurements in all patients tested. A total of 98 patients took oral hypoglycemic agents and/or received insulin treatment, while 107 patients took antihypertensive agents.

Age- and gender-matched non-diabetic controls (n=63) were randomly selected from the Shanghai TCM-Integrated Hospital for a comprehensive medical check-up. They fulfilled the following inclusion criteria: normal glucose tolerance [fasting plasma glucose <6.0 mmol/l and glycated hemoglobin (HbA_1c_; A1C) <6%]; normal blood pressure [systolic blood pressure (SBP) <140 mmHg and diastolic blood pressure (DBP) <90 mmHg]; u-ACR <30 mg/g Cr; eGFR >60 ml/min/1.73 m^2^; serum creatinine <1.2 mg/dl; no prior history of diabetes or of renal or cardiovascular diseases.

### Measurements

Fasting plasma and random spot urine were collected from subjects at their clinical visits when the anthropometric measurements were performed. Medical histories were obtained from direct interview with the patients. Concentrations of laboratorial variables, such as urinary/serum creatinine and HbA_1c_, were measured using conventional laboratory techniques. Urine aliquots were stored at −80°C prior to being used for measurements of urinary markers. The urinary concentration of TNF-α was determined using an enzyme-linked immunosorbent assay (ELISA) with a Human TNF-α Quantikine ELISA kit (DTA00C; R&D systems, Minneapolis, MN, USA), while that of NGAL was measured with a Human NGAL ELISA kit (KIT036; Thermo Fisher Scientific Inc., Rockford, IL, USA), in accordance with the manufacturer's instructions. The urine levels of the biomarkers were normalized to the urinary creatinine concentration to control for variations in hydration status.

The patients with diabetes (n=125) who had an eGFR of >60 ml/min/1.73 m^2^ and with normo- or microalbuminuria were subsequently followed-up for 28 (25–32) months with routine measurements of creatinine and UAE. The Modification of Diet in Renal Disease (MDRD) formula was used to calculate the eGFR in Chinese people as follows: MDRD = 186 × [serum creatinine (mg/dl)]^−1.154^ × (age in years)^−0.203^ × 0.742 (if female) (25). From the follow-up measurements of eGFR, linear regression was used to calculate the annual changes in eGFR.

### Statistical analysis

Normally distributed data are expressed as the mean ± standard deviation or as a percentage, while non-normally distributed values are presented as the median (interquartile range). Logarithm-transformed values of urinary albumin, TNF-α and NGAL levels were used for analyses due to their skewed distribution. Variables between different groups were compared using analysis of variance (ANOVA), followed by Bonferroni's test for normally distributed values and by the Kruskal-Wallis test for nonparametric values. Uni- or multivariate linear regression analyses were used to determine the correlation between urinary biomarkers and albumin or decline in eGFR, respectively. In multivariate models, urinary markers were adjusted for progression promoters, as indicated in each table, or all the clinical parameters tested. Patients were divided into tertiles based on levels of TNF-α or NGAL, in order to evaluate the decline in eGFR in each group. P<0.05 was considered to indicate a statistically significant difference (two-tailed). Data were analyzed using SPSS version 15.0 statistical software (SPSS, Inc., Chicago, IL, USA).

## Results

### Baseline clinical characteristics

**Figure 1:**
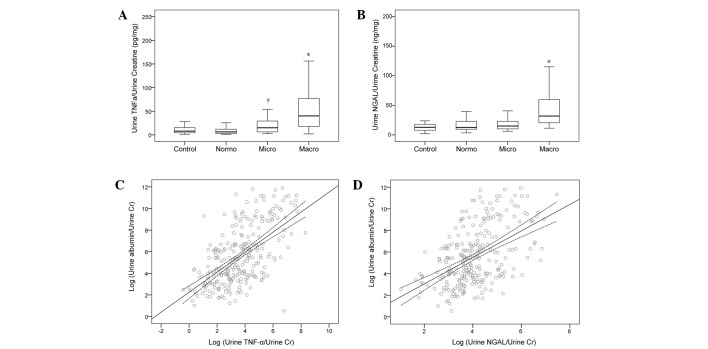
(A) Box plot graph of urine TNF-α/urine creatinine (u-TCR) measured in controls and patients with diabetes, stratified according to albuminuria status. (B) Box plot graph of urine NGAL/urine creatinine (u-NCR) measured in controls and patients with diabetes, stratified according to albuminuria status. (C) Correlation between urine albumin/urine creatinine (u-ACR) and u-TCR in patients with diabetes. (D) Correlation between u-ACR and u-NCR in patients with diabetes. Logarithm-transformed values were used for single regression analysis.

The baseline clinical characteristics of the 63 non-diabetic control and 201 patients with type 2 diabetes, stratified according to u-ACR levels, are shown in Table I. The control group and different diabetic groups were well matched with regard to age, gender and body mass index (BMI). Patients diagnosed with microalbuminuria and macroalbuminuria were more likely to have greater SBP and a longer duration of diabetes. As expected, higher levels of HbA_1c_ were observed in diabetic groups than the control. However, no significant differences were observed with regard to total cholesterol (TCHOL), low-density lipoprotein (LDL), high-density lipoprotein (HDL) or triglycerides, except that the triglyceride levels were significantly higher in the macroalbuminuria group, while HDL levels were significantly lower in the normo- and microalbuminuria groups than in the control group. Notably, eGFR levels were significantly decreased in the microalbuminuria and macroalbuminuria groups, indicating reduced kidney function in these patients. Moreover, urine TNF-α/urine creatinine (u-TCR) levels were significantly elevated in the macro- and microalbuminuria groups compared with those in the normoalbuminuria and control groups (Fig. 1A). Urine NGAL/urine creatinine (u-NCR) levels were significantly increased in the macroalbuminuria group, whereas no significant differences were observed between the normoalbuminuria, microalbuminuria and control groups (Fig. 1B). In addition, it was observed that hypoglycemic and antihypertensive agents were more widely used in the micro- and macroalbuminuria groups.

### TNF-α and NGAL are independently correlated with albuminuria in patients with type 2 diabetes

A multivariable linear regression analysis was performed to investigate the correlation between u-ACR and different markers, with u-ACR as a dependent variable. It was observed that high levels of u-TCR and u-NCR were significantly correlated with u-ACR (β=0.628, P<0.001; β=0.493, P<0.001, respectively) even after adjusting for variables known to be associated with high u-ACR (β=0.526, P<0.001; β=0.382, P<0.001, respectively) or for all clinical parameters tested (β=0.482, P<0.001; β=0.339, P<0.001, respectively; Table II). In addition, consistent with previous studies, it was observed that the duration of diabetes, baseline eGFR and HbA_1c_ were independently correlated with u-ACR under different models (Table II). No significant correlation was observed between SBP and u-ACR, which was most likely due to the frequent use of antihypertensive agents. These results revealed that urinary TNF-α and NGAL levels were elevated and independently correlated with albuminuria status, indicating that they had the potential to be used as DN markers.

### High TNF-α and NGAL levels are correlated with a greater decline in eGFR

**Figure 2:**
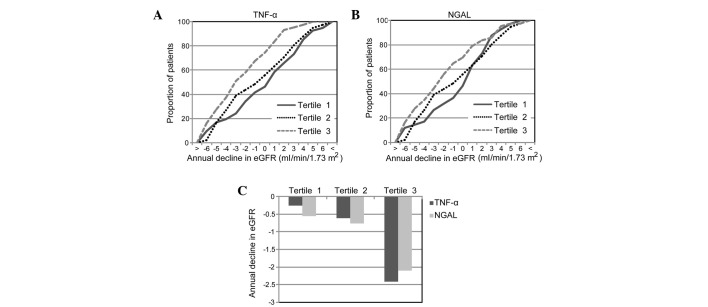
Cumulative percentage of patients with diabetes, allowing for different cut-offs of annual decline in estimated glomerular filtration rate (eGFR), in three groups stratified according to their baseline levels of (A) urine TNF-α/urine creatinine (u-TCR) or (B) urine NGAL/urine creatinine (u-NCR), respectively. Protein levels are lowest in tertile 1 and highest in tertile 3. (C) Mean baseline levels of u-TCR and u-NCR in different tertiles.

A decline in the eGFR was commonly observed and closely correlated with the progression of DN in patients with type 2 diabetes. To investigate the predictive value of u-TCR and u-NCR for DN progression at an early stage, 125 patients with diabetes with no signs of severe renal damage (eGFR >60 ml/min/1.73 m^2^ with normo/microalbuminuria) were followed up for 28 (25–32) months. During the follow-up, a median annual decline in eGFR of −1.03 ml/min/1.73 m^2^ was observed. Notably, it was identified that patients with u-TCR in the highest tertile were more likely to have a faster decline in eGFR than patients in the lowest tertile (−2.42 vs. −0.26, P=0.008, Students' t-test) or in the middle tertile (−2.42 vs. −0.62, P=0.016, Students' t-test), whereas no significant difference was observed between the middle and lowest tertiles (−0.62 vs. −0.32, P=0.617, Students' t-test) (Fig. 2A and C). Compared with the expected occurrence due to chance, patients with a u-NGAL in the highest tertile were more likely to have a greater decline in eGFR than patients in the lowest tertile (−2.11 vs. −0.55, P=0.056, Students' t-test). However, no significant difference was observed between patients in the highest and middle tertiles (−2.11 vs. −0.76, P=0.118, Students' t-test) or between the middle and lowest tertiles (−0.76 vs. −0.55, P=0.611, Students' t-test). These results indicate that high levels of baseline u-TCR and u-NCR were correlated with a rapid decline in eGFR in patients with diabetes.

### TNF-α levels are independently predictive of a decline in eGFR

It has been demonstrated that albuminuria is one of the strongest predictors of eGFR decline during the development of DN (6,10). Therefore, it was important to test whether TNF-α and NGAL were able to provide additional prognostic information concerning DN after controlling for the influence of albuminuria and other risk factors. Thus, we performed a multivariable linear regression analysis, with annual decline in eGFR as a dependent variable (Table III). It was observed that u-TCR (β=0.302, P<0.001) and u-NCR (β=−0.213, P=0.017) alone were significantly correlated with a decline in the eGFR (Table III). Subsequent to adjusting for known progression promoters, such as albuminuria, age and HbA_1c_, increased u-TCR levels remained significantly correlated with a rapid decline in eGFR (β=−0.212, P=0.024). By contrast, the influence of u-NCR was not independent of other progression promoters (β=−0.113, P=0.219). Similar results were observed even if adjustments were made for all clinical parameters tested (u-TCR, β=−0.256, P=0.012; u-NCR, β=−0.099, P=0.301). These results are consistent with prior observations that TNF-α is important in the promotion of renal injury and the disruption of the glomerular permeability barrier. Accordingly, changes in TNF-α level may precede the development of microalbuminuria in at least a subset of patients. It was thus suggested that TNF-α was a sensitive, independent predictor for the early stage of DN.

## Discussion

DN is a heterogeneous disease in which almost every aspect of the renal structure and function may be disrupted (1,4,5). Various mechanisms that lead to the pathological changes in the diabetic kidney have been proposed and are actively being investigated. For example, it has been shown that hyperglycemia may lead to increased oxidative stress and the activation of a number of inflammatory and apoptotic pathways in the kidney, resulting in podocyte malfunction and an excessive accumulation of extracellular matrix in the glomerulus and tubulointerstitium (6,8,10,26,27). It has also been proposed that high glomerular blood flow is likely to cause glomerular capillary distention, as well as glomerular and mesangial cell dysfunction (21,23). Furthermore, there have been indications that the dysregulation of signaling cascades involving a number of cytokines and growth factors, such as protein kinase C (PKC) and transforming growth factor-β (TGF-β), is also detrimental in glomerular hyperperfusion and hyperfiltration (5,23,26,27).

Clinically, glomerular injury is considered to be an early sign of DN and microalbuminuria is a strong predictor of DN progression (6,10). However, it has increasingly been suggested that many patients with normoalbuminuria are likely to develop DN (17), while not all patients with proteinuria are likely to develop progressive renal dysfunction (14,16,18). This indicates that different mechanisms may contribute to the pathogenesis of the disease. Multiple structural and pathological changes may occur concurrently and progress at different rates in the diabetic kidney, leading to the high heterogeneity of the disease. Accordingly, biomarkers with a high specificity to different abnormalities are required to predict the onset and progression of DN in different subsets of patients (13,28). Recently, a number of potential biomarkers have been identified, including markers of glomerular injury, tubular injury, inflammation and oxidative stress (5). However, there remains a requirement for the sensitivity and specificity of these markers to be compared with albumin and each other.

In this study, we investigated the correlation of the urinary inflammatory marker TNF-α and the tubular marker NGAL with albuminuria as well as their clinical applicability in predicting a decline in kidney function. Consistent with previous studies (9,24), it was demonstrated that TNF-α and NGAL levels were significantly elevated and correlated with the severity of albuminuria in patients with diabetes. During the follow-up, it was observed that albuminuria was the strongest independent predictor for eGFR decline, indicating that glomerular injury was one of the most important pathological changes in early DN. Notably, it was revealed that high urinary levels of TNF-α were significantly correlated with a rapid eGFR decline over time, even subsequent to adjustment for other progression promoters. By contrast, the influence of NGAL on eGFR decline was independent of albuminuria. Highly correlated NGAL and albuminuria levels indicated that glomerular injury and tubular injury may occur concurrently in these patients. However, at least in a subset of patients, kidney inflammation may occur prior to the onset of microalbuminuria and be causally linked to a decline in kidney function. This is consistent with previous studies, which suggested that TNF-α may be important in the pathogenesis of DN by promoting inflammation, apoptosis and oxidative stress in the diabetic kidney (23,24).

During the follow-up, five patients (8.3%) with microalbuminuria progressed to macroalbuminuria. Notably, our results showed that urinary TNF-α (log transformed TCR) levels were significantly higher in these patients than in the patients who did not develop macroalbuminuria (5.42±0.52 vs. 3.64±1.04, P=0.008, Students' t-test). However, no significant differences were observed for NGAL (4.05±0.78 vs. 4.02±1.02, P=0.948, Students' t-test). In the logistic regression analysis, baseline albuminuria (P=0.129), TNF-α (P=0.135) and NGAL (P=0.803) were not significantly correlated with macroalbuminuria. Although these results should be interpreted with caution due to the small sample size of the patients who progressed to macroalbuminuria, the role of TNF-α as an early predictor for the development and progression of DN may still be emphasized. These results were consistent with inflammation being crucial in promoting the development and progression of DN (5,21,29).

Advances in the proteomic-based biomarker discovery approach have greatly facilitated the identification of novel markers of DN (5,13,30,31). However, the progression of DN in patients with diabetes is complex and highly variable between different individuals. Accordingly, there is still a requirement for the clinical applicability of these markers to be validated in more longitudinal studies (1,5). Using a panel of biomarkers representing different pathological changes, instead of using albumin alone, appears to be a desirable approach for the early diagnosis of DN. However, more sensitive assays with high accuracy and reproducibility are required. A number of novel approaches, including targeted proteomics [multiple reaction monitoring (MRM) assay], have been successfully shown to efficiently quantify multiple biomarkers simultaneously from the same sample with high accuracy (32). This is likely to facilitate the diagnosis and treatment of early DN to prevent the long-term devastating outcomes of renal loss.

In conclusion, our results showed that urinary levels of the proinflammatory marker TNF-α and the tubular marker NGAL were elevated and correlated with albuminuria in patients with type 2 diabetes. Moreover, elevated levels of urinary TNF-α and NGAL were predictive of a decline in eGFR. Following adjustment for other progression promoters, a high TNF-α level remained an independent predictor of a rapid eGFR decline. These results suggest that TNF-α may be significant in the pathogenesis and progression of the early stage of DN. An enhanced understanding of the inflammatory response in the diabetic kidney may be beneficial to facilitate the identification of novel therapeutic strategies for the treatment of DN.

## Figures and Tables

**Table I:** Baseline characteristics of controls and patients with type 2 diabetes, stratified according to albuminuria status.

Characteristic	Control	Normoalbuminuria	Microalbuminuria	Macroalbuminuria	P-value
No. of patients	63	77	72	52	
Age (years)	61.95±9.98	60.27±11.87	62.25±10.99	63.63±11.88	0.409
Gender (male/female)	29/34	43/34	42/30	23/29	0.093
BMI (kg/m^2^)	23.04±3.81	23.65±3.78	23.35±4.17	22.04±4.15^d^	0.143
Duration of diabetes (years)	-	5.25±5.31	7.07±4.13^d^	8.94±3.87^d^,^f^	0.003
SBP (mmHg)	119.35±9.15	122.32±16.52	125.43±9.84^b^	126.33±12.10^b^	0.011
DBP (mmHg)	74.76±8.89	78.33±10.88	77.54±8.02	80.46±9.41^b^	0.013
HbA_1c_ (%)	6.05±2.27	7.01±1.89^c^	7.42±2.46^c^	7.61±2.42^c^	<0.001
TCHOL	201.65±25.94	190.87±48.17	191.24±48.17	200.48±47.34	0.246
Triglyceride (mg/dl)^a^	123.3 (87.8–159.9)	131.2 (91.2–187.5)	138.3 (109.7–173.4)	141.9 (105.1–189.8)^b^	0.358
LDL (mg/dl)	116.43±27.97	113.92±41.53	110.45±28.64	115.27±42.41	0.605
HDL (mg/dl)	59.06±20.41	48.5±16.23^b^	50.83±14.96^b^	53.91±17.42	0.053
eGFR (ml/min/1.73 m2)	97.31±19.95	90.36±17.5^b^	89.67±16.47^c^	73.82±18.09^c^,^e^,^f^	<0.001
u-ACR (ng/mg)^a^	15.1 (8.9–25.7)	11.3 (5.9–17.1)	73.5 (45.8–103.4)^c^,^e^	729.1 (545.5–1703.1)^c^,^e^,^g^	<0.001
u-TCR (pg/mg)^a^	8.04 (4.38–15.74)	6.21 (2.82–12.12)	15.09 (6.29–29.31)^b^,^e^	40.42 (18.47–77.18)^c^,^e^,^g^	<0.001
u-NCR (ng/mg)^a^	12.82 (6.52–19.62)	12.54 (8.08–27.61)	14.99 (8.7–25.88)^b^	32.03 (21.11–63.71)^c^,^e^,^g^	<0.001
Hypoglycemic agents, n (%)	-	23 (29.9)	46 (63.9)^e^	29 (55.8)^e^	<0.001
Antihypertensive agents, n (%)	-	15 (19.5)	58 (80.6)^e^	34 (65.4)^e^	<0.001

Data are expressed as the mean ± standard deviation or as percentages for normal distribution. Non-normally distributed values are presented as median (interquartile range).

a Logarithm-transformed values were used for comparison;

b P<0.05 vs. control;

c P<0.001 vs. control;

d P<0.05 vs. normoalbuminuria;

e P<0.001 vs. normoalbuminuria;

f P<0.05 vs. microalbuminuria;

g P<0.001 vs. microalbuminuria.

BMI, body mass index; SBP, systolic blood pressure; DBP, diastolic blood pressure; HbA_1c_, hemoglobin A1c; TCHOL, total cholesterol; LDL, low-density lipoprotein; HDL, high-density lipoprotein; eGFR, estimated glomerular filtration rate; u-ACR, urine albumin/urine creatinine; u-TCR, urine TNF-α/urine creatinine; u-NCR, urine NGAL/urine creatinine.

**Table II:** Multiple linear regression analyses of u-ACR as a dependent variable in patients with type 2 diabetes.

	u-TCR			u-NCR		
		
Variable	Model 1	Model 2	Model 3	Model 1	Model 2	Model 3
Marker	0.628 (<0.001)	0.526 (<0.001)	0.482 (<0.001)	0.493 (<0.001)	0.382 (<0.001)	0.339 (<0.001)
Age		0.001 (0.980)	0.078 (0.938)		0.017 (0.768)	0.025 (0.666)
Duration		0.198 (<0.001)	0.182 (<0.001)		0.205 (<0.001)	0.187 (0.002)
HbA_1c_		0.092 (0.076)	0.081 (0.122)		0.146 (0.011)	0.122 (0.031)
eGFR		−0.251 (<0.001)	−0.241 (<0.001)		−0.274 (<0.001)	−0.246 (<0.001)
SBP		−0.013 (0.808)	−0.017 (0.767)		0.032 (0.576)	−0.03 (0.633)
DBP			0.039 (0.541)			0.114 (0.095)
Gender			0.019 (0.711)			0.005 (0.93)
BMI			−0.087 (0.107)			−0.13 (0.027)
TCHOL			0.027 (0.653)			0.014 (0.827)
Triglyceride			−0.021 (0.741)			0.065 (0.315)
LDL			-			-
HDL			−0.014 (0.831)			0.007 (0.919)
Hypoglycemic agents			0.090 (0.086)			0.045 (0.426)
Antihypertensive agents			0.126 (0.024)			0.154 (0.013)

Correlation between different variables and baseline urine albumin/urine creatinine (u-ACR) are shown as standard β (P-value). Log transformed values of u-ACR, u-TCR and u-NCR were used. u-TCR, urine TNF-α/urine creatinine; u-NCR, urine NGAL/urine creatinine; model 1, unadjusted; model 2, adjusted for variables known to be associated with high u-ACR; model 3, adjusted for all variables tested; HbA_1c_, hemoglobin A1c; eGFR, estimated glomerular filtration rate; SBP, systolic blood pressure; DBP, diastolic blood pressure; BMI, body mass index; TCHOL, total cholesterol; LDL, low-density lipoprotein; HDL, high-density lipoprotein.

**Table III:** Multiple linear regression analyses of the annual decline in eGFR as a dependent variable in patients with type 2 diabetes.

	u-TCR			u-NCR		
		
Variable	Model 1	Model 2	Model 3	Model 1	Model 2	Model 3
Markers	−0.302 (<0.001)	−0.212 (0.024)	−0.256 (0.012)	−0.213 (0.017)	−0.113 (0.219)	−0.099 (0.301)
u-ACR		−0.228 (0.017)	−0.243 (0.017)		−0.274 (0.004)	−0.295 (0.004)
Age		−0.05 (0.57)	−0.066 (0.482)		−0.036 (0.688)	−0.066 (0.494)
Duration of diabetes		0.035 (0.691)	0.02 (0.827)		0.024 (0.788)	−0.006 (0.947)
HbA_1c_		−0.058 (0.507)	−0.049 (0.581)		−0.078 (0.375)	−0.065 (0.474)
eGFR		0.128 (0.143)	0.13 (0.14)		0.105 (0.239)	0.104 (0.246)
SBP		0.025 (0.769)	−0.03 (0.777)		0.02 (0.825)	−0.004 (0.97)
DBP			0.111 (0.363)			0.032 (0.792)
Gender			−0.055 (0.546)			−0.018 (0.847)
BMI			−0.158 (0.108)			−0.097 (0.329)
TCHOL			-			-
Triglyceride			−0.035 (0.728)			−0.062 (0.548)
LDL			−0.137 (0.153)			−0.164 (0.091)
HDL			0.02 (0.848)			0.029 (0.788)
Hypoglycemic agents			−0.117 (0.2)			−0.098 (0.295)
Antihypertensive agents			0.184 (0.064)			0.125 (0.204)

Correlation between different variables and the annual decline in eGFR are shown as standard β (P-value). Log transformed values of u-ACR, u-TCR and u-NCR were used. eGFR, estimated glomerular filtration rate; model 1, unadjusted; model 2, adjusted for variables known to be associated with fast eGFR decline; model 3, adjusted for all variables tested; u-TCR, urine TNF-α/urine creatinine; u-NCR, urine NGAL/urine creatinine; model 1, unadjusted; model 2, adjusted for variables known to be interrelated with high u-ACR; model 3, adjusted for all variables tested; u-ACR, urine albumin/urine creatinine; HbA_1c_, hemoglobin A1c; SBP, systolic blood pressure; DBP, diastolic blood pressure; BMI, body mass index; TCHOL, total cholesterol; LDL, low-density lipoprotein; HDL, high-density lipoprotein.
